# Plasmacytoid Dendritic Cell Dynamics Tune Interferon-Alfa Production in SIV-Infected Cynomolgus Macaques

**DOI:** 10.1371/journal.ppat.1003915

**Published:** 2014-01-30

**Authors:** Timothée Bruel, Stéphanie Dupuy, Thomas Démoulins, Christine Rogez-Kreuz, Jacques Dutrieux, Aurélien Corneau, Antonio Cosma, Rémi Cheynier, Nathalie Dereuddre-Bosquet, Roger Le Grand, Bruno Vaslin

**Affiliations:** 1 Division of Immuno-Virology, Institute of Emerging Diseases and Innovative Therapies, CEA, Fontenay-aux-Roses, France; 2 Unité Mixte de Recherche UMR-E01, Université Paris-Sud, Orsay, France; 3 Neurovirology Laboratory, Bertin Pharma, Fontenay-aux-Roses, France; 4 Université Paris Descartes, Sorbonne Paris Cité, Paris, France; 5 INSERM, U1016, Institut Cochin, Paris, France; 6 CNRS, UMR8104, Paris, France; 7 Université Paris Diderot, Paris, France; Emory University, United States of America

## Abstract

IFN-I production is a characteristic of HIV/SIV primary infections. However, acute IFN-I plasma concentrations rapidly decline thereafter. Plasmacytoid dendritic cells (pDC) are key players in this production but primary infection is associated with decreased responsiveness of pDC to TLR 7 and 9 triggering. IFNα production during primary SIV infection contrasts with increased pDC death, renewal and dysfunction. We investigated the contribution of pDC dynamics to both acute IFNα production and the rapid return of IFNα concentrations to pre-infection levels during acute-to-chronic transition. Nine cynomolgus macaques were infected with SIVmac251 and IFNα-producing cells were quantified and characterized. The plasma IFN-I peak was temporally associated with the presence of IFNα^+^ pDC in tissues but IFN-I production was not detectable during the acute-to-chronic transition despite persistent immune activation. No IFNα^+^ cells other than pDC were detected by intracellular staining. Blood-pDC and peripheral lymph node-pDC both lost IFNα^−^ production ability in parallel. In blood, this phenomenon correlated with an increase in the counts of Ki67^+^-pDC precursors with no IFNα production ability. In tissues, it was associated with increase of both activated pDC and KI67^+^-pDC precursors, none of these being IFNα^+^ *in vivo*. Our findings also indicate that activation/death-driven pDC renewal rapidly blunts acute IFNα production *in vivo*: pDC sub-populations with no IFNα-production ability rapidly increase and shrinkage of IFNα production thus involves both early pDC exhaustion, and increase of pDC precursors.

## Author Summary

Chronic immune activation is a characteristic of HIV infection and a key contributor to CD4 T-cell depletion and progression to AIDS. Persistent up-regulation of interferon-induced genes (ISG) is associated with chronic immune activation and is a molecular signature of the progression of SIV infection in non-human-primate models. Nevertheless, the type and tissue compartmentalization of IFN-I-producing cells at different stages of infection, and the details of the involvement of IFN-I in sustaining chronic immune activation remain elusive. Using the cynomolgus macaque model of progressive SIV infection, we demonstrate *in vivo* that plasmacytoid dendritic cells (pDC) are major contributors to IFNα production in lymphoid tissues and, most importantly, that this production rapidly shrinks after primary infection. IFNα production rapidly decreased as a consequence of both activation-induced exhaustion of pDC, and their replacement by pDC precursors with no IFNα production ability. Our data indicate that pDC renewal contributes to the rapid contraction of pDC-derived IFNα production during primary infection, which may favor the transition from acute-to-chronic infection by limiting the efficacy of innate immunity.

## Introduction

HIV-1 infection is characterized by chronic immune activation, a major cause of CD4 T-cell depletion and HIV/SIV-specific immunity dysfunction, and facilitating viral replication and progression to AIDS [1]. Simian immunodeficiency virus (SIV) infection in non human primates (NHP) leads to chronic immune activation and AIDS in macaques, but not in the natural African NHP hosts despite persistently high viremia [2]. Strong expression of interferon-stimulated genes (ISGs) in chronic infection distinguishes pathogenic from non-pathogenic models; this suggests that control of IFN-I responses is critical for HIV/SIV pathogenesis [2], [3], [4], [5]. Unraveling the underlying mechanisms of IFN-I induction and control may therefore reveal novel possibilities for new therapeutic strategies.

Acute interferon-alpha (IFNα) production is observed in both lymphoid and non-lymphoid tissues during primary Simian Immunodeficiency Virus (SIV) infection (PSI) [6], [7], but is barely detectable during the chronic stage of pathogenic HIV/SIV infection until the late symptomatic stage [4], [8], [9], [10]. The cellular source of IFN-I and site of its activity during the early chronic phase remain elusive, and the mechanism leading to the reduction of IFNα production during the acute-to-chronic transition phase of HIV/SIV infections have not been rigorously described [11].

Plasmacytoid dendritic cells (pDC) are bone marrow (BM)-derived antigen-presenting cells that are central to innate and adaptive immunity [12], [13]. They selectively express Toll-like receptors (TLR) 7 and 9 and their constitutive expression of interferon response factor 7 (IRF-7) makes them major IFN-I producing cells in response to viruses.

*In vitro*, pDC are activated by HIV/SIV particles [14], [15] and produce IFNα following sensing by TLR-7 [16], [17]. A small number of studies show that IFNα is produced *in vivo* by pDC: in the vaginal mucosa early after exposure [18] and in LN during acute infection [19] in SIV macaque models; and advanced chronic infection in HIV-1 infected patients [20], [21], [22]. During the chronic phase, other cell types in the spleen may also produce IFNα [23].

In contrast, pDC are quantitatively and functionally affected by HIV/SIV infection. During HIV infection pDC counts correlate negatively with viremia [24] and are predictive of progression [25], [26]. In Non Human Primates (NHP), pDC counts in blood decline, and this is inversely correlated with their recruitment in LN as early as during acute infection [5], [27]. In these tissues, pDC may play an important role in viral control and immune regulation, but there is a massive pDC death by apoptosis [27]. More recently, it was reported that HIV/SIV infection induces a rapid and long-lasting accumulation of pDC in the gut [28], [29] where they may contribute to inflammation and chronic immune activation. Conversely, the peripheral blood pDC pool becomes less able to produce IFNα in response to *de novo* re-stimulation with SIV and HSV [5], although this dysfunction partly recovers during the acute-to-chronic transition. A transient unresponsive state has also been observed during primary HIV-1 infection [30] and in late stage HIV-1 infection [31], and was suggested to be a consequence of a refractory stage acquired following pDC activation *in vivo* [32]. These observations contrast with *in vitro* data showing that stimulation of human pDC by HIV leads to persistent IFNα production and the acquisition of a partial activation phenotype, but not a refractory stage, as a result of HIV trafficking through a specific intra-cellular pathway in these cells [33].

Plasmacytoid DC turnover is increased during acute infection [27], [34] and this may contribute to the apparent dysfunction as a result of homeostatic processes. The human BM-pDC pool includes at least three sub-populations that produce no or little IFNα upon CpG stimulation [35]; these pools probably correspond to different stages of pDC precursors. The peripheral pDC pool seems to be mainly reconstituted by Ki67^+^ BM-derived pDC precursors during acute infection [27]. Therefore, we hypothesized that this homeostatic process may directly affect pDC activation and IFNα production in the periphery during SIV infection and may play a role in the observed rapid shrinkage of acute IFNα production.

In this study, we investigated the involvement of pDC in IFNα production in blood and tissues during the early stages of SIV infection in cynomolgus macaques (CyM) and studied the role of pDC sub-population dynamics in IFNα production. We report that pDC are major cell type responsible for the massive IFNα production during acute infection in both lymphoid tissues and gut in this species, and show that pDC dynamics, including both activation-driven exhaustion and increased renewal by bone marrow derived pDC precursors with no IFNα production capacity, accounts for the rapid decline of pDC responsiveness and consequent shut-down of acute IFNα production.

## Results

### Primary infection of CyM with SIVmac251 is characterized by a transient increase in the plasma IFNα concentration and by dynamic changes in pDC blood counts with no signs of activation

**Figure 1:**
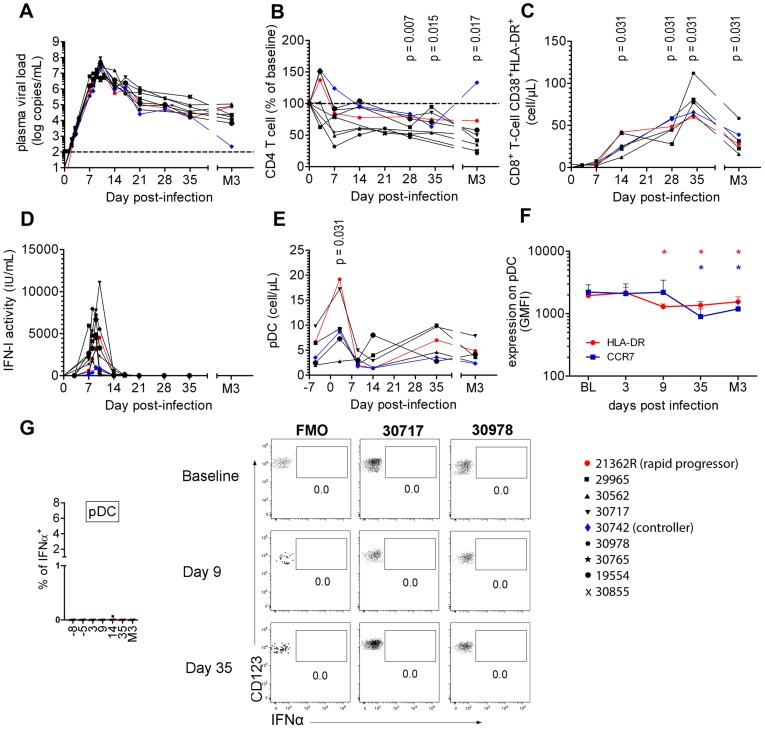
Cynomolgus macaques were infected intravenously with 5,000AID50 of SIVmac251 and chronic infection established. (**A**) Longitudinal follow-up of viral RNA load in plasma (n = 9). (**B**) CD4^+^ T-cell blood counts at various times following infection (n = 9). (**C**) Immune activation measured as HLA-DR^+^CD38^+^ co-expression by CD8^+^ T-cells (n = 6). (**D**) Type I Interferon (IFN-I) antiviral activity measured as the inhibition of Vesicular Stomatitis Virus cytotoxicity to Maddin-Darby Bovine Kidney cells (n = 9). (**E**) pDC counts in blood at various times(n = 6). (**F**) CCR7 and HLA-DR MFI on blood pDC (n = 6). (**G**) IFNα expression after various times of SIV infection in 6 macaques in defined blood cell populations, including from left to right, CD14^+^ monocytes, mDC, B cells, NK cells, CD4^+^ T cells, CD8^+^ T cells and pDC.

Nine CyM were infected with SIVmac251 and followed longitudinally (Table S1 and Figure S1). The viral RNA (vRNA) load peaked on day 9 or 10 p.i. at a mean 7.33±0.452 Log copies/mL (Figure 1A). Mean setpoint viremia was 4.21±0.815 Log copies/mL and one of the nine animals (Macaque #30742) displayed low setpoint viremia (2.34 Log copies/mL). CD4^+^ T-cell counts on day 28 were significantly below baseline levels (mean: 72.23%±12.57% of baseline) (Figure 1B) with CD4^+^ central memory T-cell counts particularly affected (mean: 52.14%±12.53% of baseline on day 28). Significant CD4^+^ T-cell count decline was observed in all animals, except #30742 (Figure 1B), and chronic CD8 T-cell immune activation (Figure 1C) was also evidenced at setpoint. Macaque #21362R was euthanized because of AIDS one year after infection (wasting syndrome, CD4 T-cells: 70 cells/µL, PVL: 5.14 Log copies/mL). The other animals remained clinically asymptomatic during this period.

There was a transient increase in the plasma IFN-I activity (Figure 1D), coinciding with the exponential increase of plasma viremia. IFN-I activities on days 7, 8 and 9 were positively correlated with plasma viral load (R = 0.631, p = 0.004) and peak values ranged from 356 to 7,942 IU/mL (mean: 3,504 IU/mL). Blood pDC counts were monitored in six macaques revealing a transient increase (mean 214±70% of baseline; p = 0.0313) on day 3 p.i. (Figure 1E, Figure S2), followed by a transient decrease on day 14 in five macaques. Plasmacytoid DC counts returned to baseline levels by day 35, and then progressively decreased in five of the six macaques, consistent with previous reports [5], [27]. Despite of these changes in the dynamics of circulating pDC, longitudinal analysis of HLA-DR and CCR7 expression levels (MFI) did not reveal any activation of circulating pDC (Figure 1F), IFNα remained undetectable by intracellular staining (Figure 1G), and IFNα mRNA expression measured be RT-qPCR in PBMC did not change at any time (data not shown).

These data show that despite dynamic changes in pDC numbers in primary infection these cells do not show any activation in the blood.

### Plasmacytoid DC strongly contribute to IFNα production in peripheral lymph nodes (PLN) during acute infection

PLN were studied in the nine SIV-infected macaques of the longitudinal study. All were sampled at baseline, three on days 7, 8 and 9 p.i., and six on days 8, 9, 35 and month 3 (M3).

**Figure 2:**
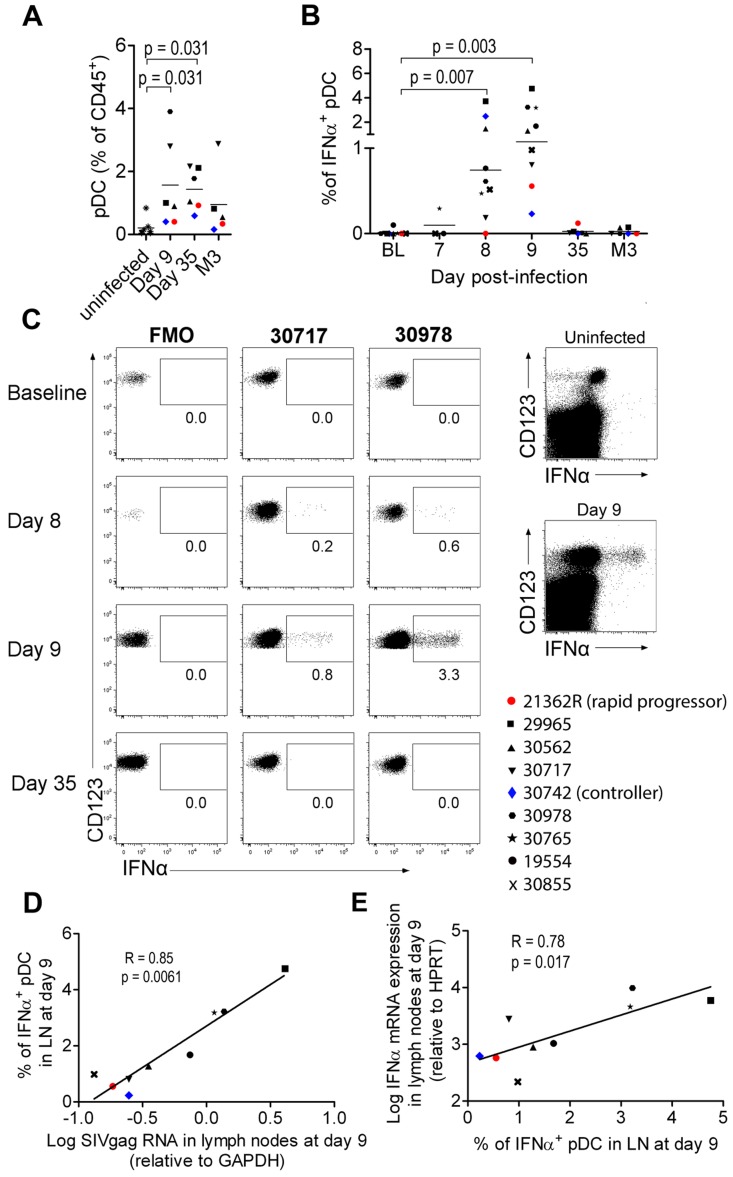
Plasmacytoid DCs are major contributors of IFNα production in peripheral lymph nodes during primary infection. (**A**) Plasmacytoid DC frequencies among CD45^+^ PLN leukocytes on days 9 and 35 and month 3 post-infection (n = 6) and in uninfected macaques (n = 7). (**B**) IFNα-producing pDC in peripheral lymph nodes of 9 macaques at various times after infection as assessed by IFNα intracellular staining. Freshly isolated cells were labeled at various times after infection without any additional *in vitro* stimulation, after 30 min incubation in the presence of 10 mg/mL Brefeldin A. (**C**) Dotplots for two representative infected macaques (#30717, #30978) with fluorescence minus one (FMO) shown as a negative control (left). Dotplot showing intracellular IFNα expression in the total live CD45^+^ leukocyte gate for one representative infected macaque at day 9 p.i., and one representative uninfected macaque (right) (**D**). The percentage of IFNα^+^ pDC correlates with relative SIVgag mRNA expression in peripheral lymph nodes (day 9 p.i., n = 9). Spearman correlation. (**E**) Log_10_ (relative IFNα mRNA expression) plotted against the percentage of IFNα expressing pDC in PLN (day 9 p.i., n = 9). Spearman correlation. Values at different time points were compared with the Wilcoxon rank sum test. When baseline values were not available, data for infected macaques were compared with uninfected macaques using the Mann-Whitney rank test; p values are given if the differences are statistically significant.

As pDC are the best candidates for the IFNα production observed, we analyzed pDC dynamics in PLN. Plasmacytoid DC counts were significantly higher in PLN of infected animals on days 9 and 35 p.i., than in uninfected macaques (Figure 2A), and a trend for higher pDC frequencies was still observed 3 months p.i.. IFNα^+^ pDC could hardly be detected in PLN at baseline by direct *ex vivo* intracellular staining, but were significantly increased in PLN on days 7, 8 (range 0% to 3.71%; p = 0.0078) and 9 (range 0.2310% to 4.754%; p = 0.0039) p.i. (Figure 2B and 2C). On and after day 35, IFNα^+^ cells could no longer be detected. This approach allowed, for the first time, quantification of IFNα-producing pDC in tissues during acute infection. To assess the contribution of viral components to induction of IFNα production by pDC, vRNA in PLN was assayed by qRT-PCR. Viral RNA peaked on day 8–9 p.i. and was significantly lower by day 35 than on day 9 (p = 0.031) (Figure S3 A). *IFNα* mRNA levels in LN correlated with viral RNA in PLN (R = 0.88, p = 0.003) (Figure S3 B). These results are consistent with the IFNα response being driven by viral components, and suggest that pDC in PLN are directly triggered by SIV vRNA. Indeed, the percentage of IFNα^+^ pDC correlated with both viral RNA load (Figure 2D) and IFNα mRNA level in whole tissue biopsies on day 9 (Figure 2E) (R = 0.783, p = 0.0172), suggesting that pDC-derived IFNα is produced in response to SIV and account for an important part of total IFNα production in PLN. Indeed, CD123^−^ leukocytes did not show any significant increase of IFNα+ compared to baseline levels (Figure 2C and data not shown). A careful analysis of DC and macrophage subpopulations (gating described in Figure S2 C) did not reveal any increase of spontaneous IFNα production by these cells in lymph nodes (data not shown).

These various findings confirm that PLN pDC are involved in IFNα production during acute SIV infection in CyM, although production at low level in this or in other cell types below the threshold of our methods cannot be excluded.

### Molecular and cellular analysis of various tissues reveals that pDC produce IFNα in both lymphoid and mucosal compartments during acute infection in CyM

**Figure 3:**
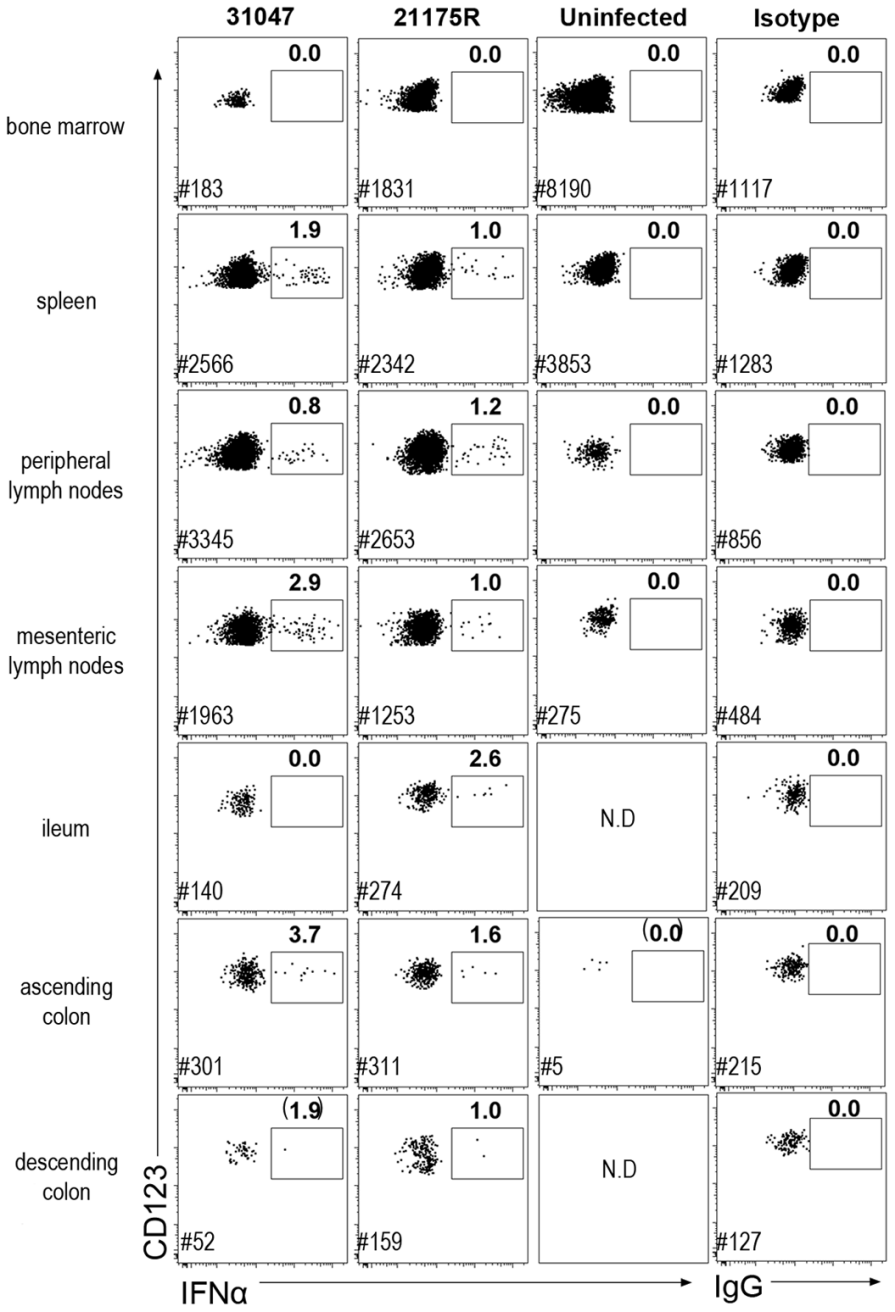
Plasmacytoid DC produce IFNα in both lymphoid and mucosal compartments. Dotplot showing IFNα intra-cellular staining in gated pDC (CD45^+^HLA-DR^+^lin^−^CD123^+^) in different tissues. Cells were labeled *ex vivo* on fresh cells after 30 min incubation in 10 µg/mL brefeldin A in the absence of any stimulation. Data for two macaques sacrificed on day 10 p.i. and one uninfected control are shown. Mononuclear cells from BM, spleen, peripheral LN, mesenteric LN, ileum, and colon were extracted for FACS analysis. Frequencies of IFNα-pDC are indicated in bold and # indicates the number of pDC recorded for each file.

The contribution of other tissues to IFNα production during acute infection was also explored in two macaques (#21175R, #31047) euthanized on day 10 p.i., and one non-infected control (cross-sectional analysis). Intracellular staining was used to track IFNα-producing cells. IFNα-producing pDC were detected in spleen, mesenteric LN, and some colon and ileum biopsies from these animals (Figure 3). The percentages of pDC that were IFNα-producers were similar to that observed in peripheral LN (0.8 to 3.7%).

Additional colon samples from 4 naïve macaques, 4 SIV-infected macaques at day 9 p.i., and 4 SIV-infected macaques in chronic infection, revealed increased pDC numbers in the colon on day 9 p.i., which persisted in chronic infection (data not shown). Remarkably, although the numbers of pDC were significantly increased in colon, numbers of pDC remained about ten fold lower than in lymph nodes. Intracellular IFNα was evidenced in colon pDC in day 9 samples, within the same range (1.5 to 4%), but were not detectable in chronic infection (data not shown).

Interestingly, in BM, which mostly contains pDC precursors that do not produce IFNα upon stimulation *in vitro* [35], intracellular IFNα was not detected in pDC. No other cell type was found to be positive for IFNα by intracellular staining in any of these tissues (data not shown).

These findings show that pDC are also major producers of systemic IFNα in various lymphoid and mucosal tissues during acute infection in CyM, and show persistent homing of pDC to the gut in the chronic phase.

### Plasmacytoid DCs display a complex activation phenotype in tissues associated with massively increased CD95 expression and death during acute infection

**Figure 4:**
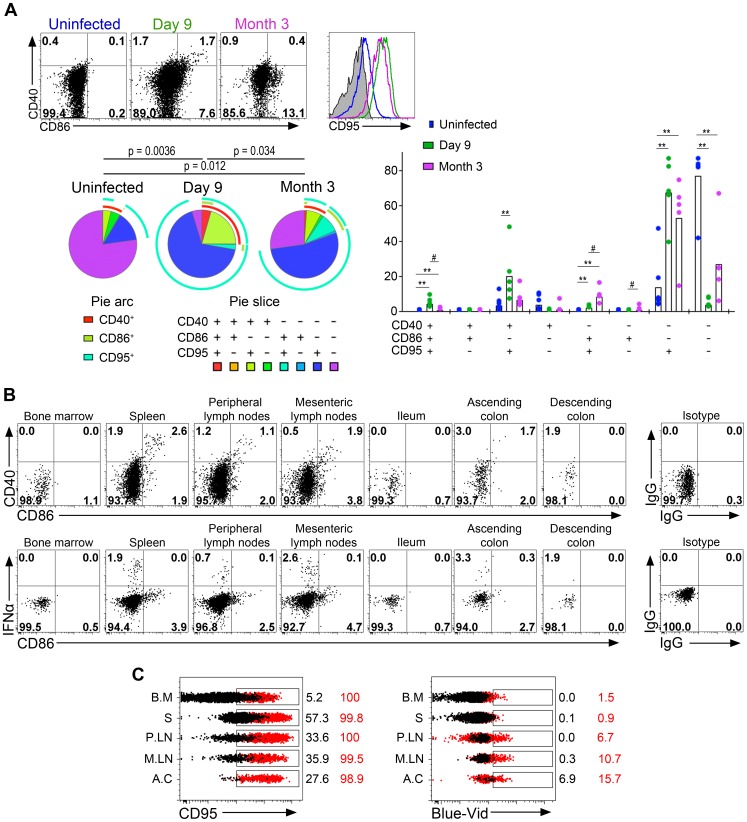
Plasmacytoid DCs in peripheral lymph nodes are strongly activated and are subject to a high death rate. (**A**) Analysis of CD40, CD86 and CD95 expression on pDC in lymph nodes of one representative uninfected macaque, and one representative infected macaque on day 9 and month 3 (M3) p.i. (**Top left**). SPICE analysis of CD40, CD86 and CD95 expressing pDC from 6 uninfected macaques and 6 infected macaques (day 9 and M3 p.i.) showing the distribution of each sub-population in total pDC as pie chart (n = 6) (**Bottom left**), and as bar chart (n = 6) (**right**) for each infection status. (**B**) Flow cytometry analysis of one animal sacrificed on day 10: CD40 and CD86 expression on gated pDC showing activated pDC with dual expression, and IFNα and CD86 expression. IFNα^+^ pDC are CD86^low/neg^. (**C**) Histogram overlays of CD95 and staining of dead cells (Blue-Vid) in various tissues (BM for bone marrow, S for spleen, PLN for peripheral lymph nodes, MLN for mesenteric lymph nodes, AC for ascending colon) from one of two sacrificed macaques (red) and one uninfected control (black).

The increase of CCR7^high^ pDC in LN led us to analyze pDC activation further. The expression of CD40, CD86 and CD95 was studied by flow cytometry (Figure 4). In non-infected macaques, 77.1±18.4% of pDC expressed none of these three markers, activated pDC (expressing either CD40 and/or CD86) made up 8.6±6% of total pDC in PLN, and CD95 was expressed by 18.3±20.1%. On day 9 p.i., 29.1±22.5% of LN pDC (range: 13.8 to 67.5%) expressed CD40 or CD86, or both, evidence of a significant increase of activation (Figure 4A). Activated pDC also showed HLA-DR^high^ expression (data not shown). An increase of CD95 expression was also observed and exceeded that of activated cells (Figure 4A). Indeed, on day 9 p.i., more than 95.0±2.9% of pDC expressed high CD95 levels. Activation of pDC and CD95 expression were both significantly reduced at setpoint but remained significantly higher than at baseline. Remarkably, dual-expression of CD40 and CD86, characteristic of the full activation phenotype, occurred *in vivo*. At setpoint, pDC showed much lower CD40/CD86 co-expression and higher monovalent CD86^+^ expression than during acute infection, suggesting that partial activation may occur in the absence of detectable IFNα. Interestingly, macaque #30742, which efficiently controlled viremia, displayed the lowest CD95 expression at setpoint suggesting that viral load may directly or indirectly sensitize pDC to apoptosis. The two macaques sacrificed on day 10 p.i. were used to explore pDC activation in other tissues and to analyze the phenotype of IFNα-producing pDC with antibodies against CD95, CD40, CD86 and IFNα. Plasmacytoid DC in all tissues in which IFNα production was observed displayed a similar activated phenotype (Figure 4B). Remarkably, no IFNα^+^ pDC were CD86^+^ (Figure 4B) or CD40^+^ (data not shown), suggesting that expression of these activation markers are later events than IFNα production, reminiscent of the kinetics we observed following TLR7/8 stimulation *in vitro* (data not shown). The large increase of CD95 expression on pDC was confirmed in all tissues explored including BM, spleen, LN and gut, and was associated with increased pDC death (Figure 4C) although the proportion of dead pDC (Blue-Vid^+^) was much lower than that of CD95^+^pDC.

Overall, the complexity of pDC phenotype in tissues suggests successive stages in pDC activation leading to immediate but transient IFNα production followed by a slower increase of the expression of activation markers, which is associated with a massive increase of both CD95 expression and cell death.

### Impaired responsiveness of circulating blood pDC during acute infection correlates with increased non-functional pDC precursor frequencies

**Figure 5:**
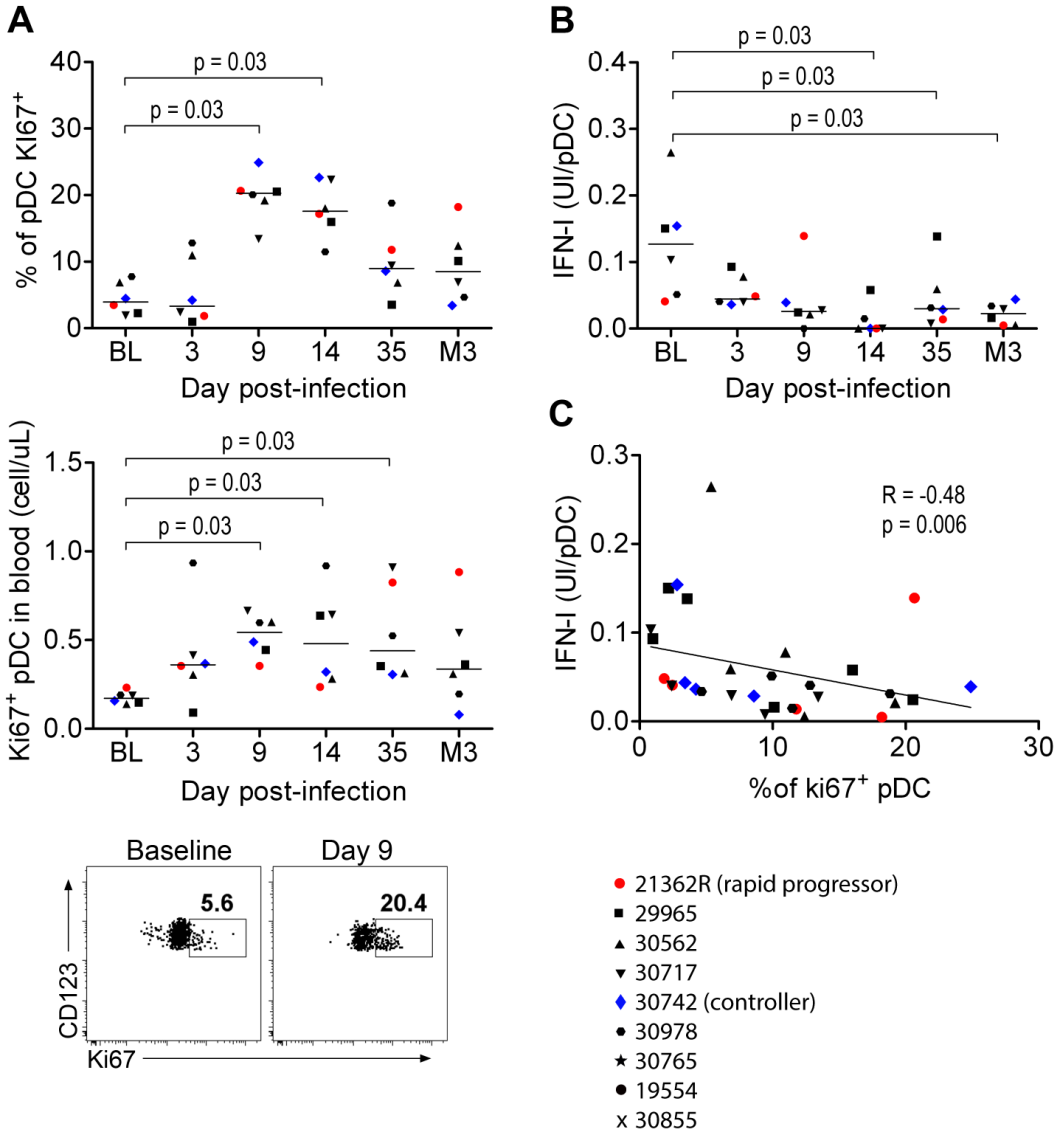
Circulating PDC show decreased responses to SIV inversely correlated with the increased prevalence of Ki67^+^ pDC precursors. (**A**) Ki67^+^ pDC precursors counts during primary infection in the blood of 6 macaques, as a percentage of the total pDC population (**Top**) and as absolute counts (**Middle**), and dot plot showing increase of Ki67^+^ pDC in one representative animal from baseline to day 9 p.i. (**Bottom**). (**B**) Evolution of IFNα production per pDC in response to stimulation with 200 ng p27 equivalent of inactivated SIV (SIV-AT-2) for 24 h (n = 6). (**C**) IFN-I production per pDC in response to SIV-AT2 correlates negatively with the Ki67^+^ pDC counts.

IFNα production by pDC was associated with increased numbers of these cells in tissues and increased death rate, suggesting high turnover. Consistent with reported increased numbers of BM-derived Ki67^+^ pDC in both peripheral blood and PLN of Rhesus Macaques (RhM) during acute infection as a consequence of LN homing [27], [34], we detected higher absolute counts and percentages of Ki67^+^pDC in the blood during acute infection than in baseline (Figure 5A). We then studied the potential contribution of the Ki67^+^ pDC increase to the altered responsiveness of circulating pDC as bone marrow pDC are weak IFNα producers upon stimulation [35], [36]. IFN-I production by blood pDC in response to SIV *in vitro* was significantly altered as early as 14 days p.i. (p = 0.031) (Figure 5B), and this defect was temporally associated with the increase in Ki67^+^ pDC counts. Moreover, the IFNα production ability of pDC was negatively correlated with the increase in Ki67^+^pDC counts in blood (R = 0.48, p = 0.006) (Figure 5C). This suggests that total pDC numbers decrease in the blood during acute infection, and are renewed by Ki67^+^ pDC egressing from the bone marrow, accounting for the decreased responsiveness of circulating pDC.

### Macaque bone marrow pDC are poor IFN-I producers

**Figure 6:**
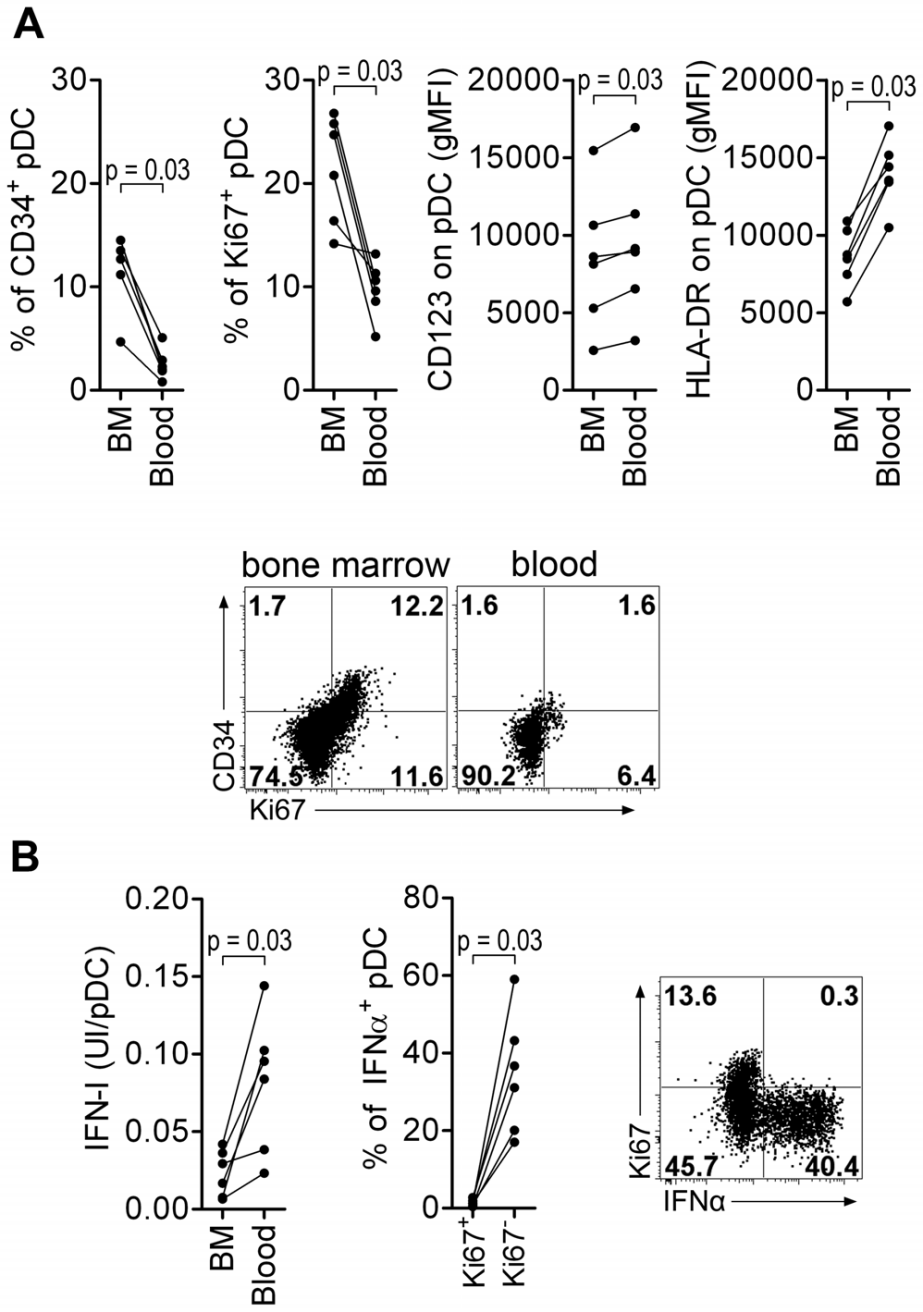
Macaque bone marrow pDC express lower CD123 and HLA-DR, display higher percentages of CD34+ and Ki67+ precursors than blood pDC, and are poor IFN-I producers. (**A**) CD34^+^ and Ki67^+^ pDC-precursor frequencies are higher in the bone marrow (BM) than in the blood (non-infected macaques, n = 6), and both CD123 and HLA-DR expressions are lower in BM pDC than blood pDC. From **left to right**: Percentage of CD34^+^ pDC precursors, percentage of Ki67^+^ pDC precursors, and CD123 and HLA-DR geometric MFI (gMFI) in BM and blood pDC. Dotplot showing Ki67 and CD34 expression by pDC in BM and blood, from one representative animal. (**B**) Bone marrow pDC produce less IFN-I in response to TLR-7/8 stimulation (R848) than blood pDC (non-infected macaques, n = 6) (**Left**). Most IFNα is produced by Ki67^−^ pDC and not by K67^+^ precursors (**Middle**). Representative dot plot showing that only Ki67^−^ pDC produce IFNα (**Right**). Wilcoxon's rank sum test was used for all comparisons of paired data.

As human BM contains several pDC precursor subpopulations with differential IFNα-production ability, we looked at the phenotype and the ability of CyM BM-pDC to produce IFNα. In non-infected CyM, the frequencies of Ki67^+^ and CD34^+^pDC among total pDC were higher in BM than in peripheral blood (Figure 6A), consistent with a recent report in humans [35]. The expression levels of HLA-DR and CD123 were also lower on BM pDC than on peripheral blood pDC (Figure 6A). Ki67^+^pDC did not produce IFNα in response to TLR7/8 ligands leading to a much lower IFNα production by BM than blood pDC (Figure 6B).

Thus, the BM-pDC pool includes several sub-populations of pDC, likely precursors as previously suggested for their human counterparts [35], which are mostly unable to produce IFNα in response to SIV stimulation. Consequently, the progressive increase of these subpopulations in blood during acute infection accounts for the observed decrease of IFNα production capacity by circulating pDC in primary infection.

### Neither pDC expressing activation markers nor late pDC precursors produce IFNα *in vivo* and the counts of both increase in lymph nodes during acute infection

**Figure 7:**
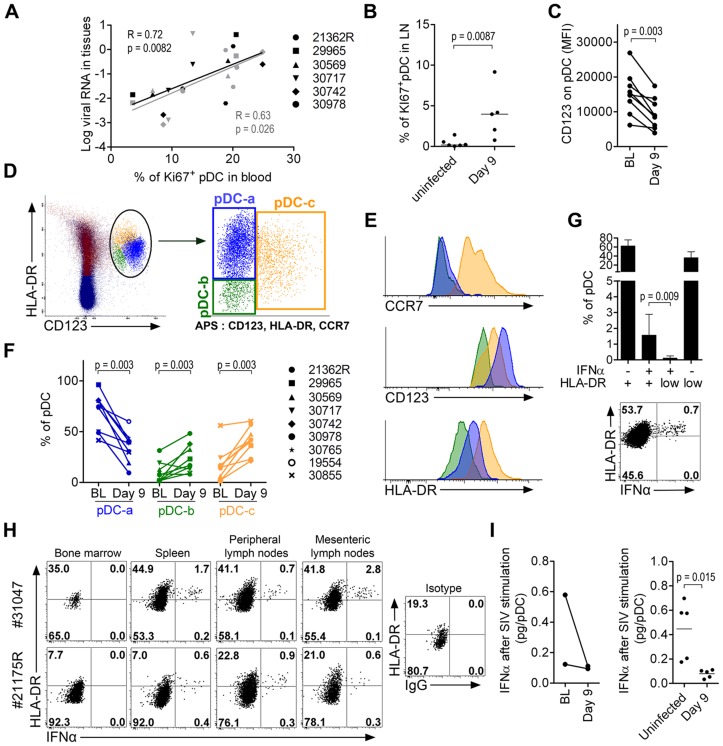
Plasmacytoid DC precursors disseminate into tissues during primary infection and are consistently IFNα negative. (**A**) Mobilization of Ki67^+^ pDC precursors to the blood correlates with tissue viral loads (LN in black and rectum in gray). Spearman correlation. (**B**) Higher Ki67^+^ expression on LN pDC in infected animals (n = 5, day 9 p.i.) than uninfected animals (n = 6). (**C**) Lower CD123 expression in LN-pDC on day 9 p.i. than at baseline (n = 9). (**D**) Principal component analysis (APS = automated population separator using Infinicyt software) of pDC based on HLA-DR, CD123, and CCR7 expression from merge files of both pre- and day 9 post-infection allowed the identification of three sub-populations in LN. (**E**) Respective HLA-DR, CD123 and CCR7 expression by the three LN pDC sub-populations. (**F**) Changes of the three LN pDC sub-populations following SIV infection (from BL to day 9 p.i., n = 9). (**G**) Frequencies of IFNα pDC in LN according to HLA-DR expression showing that IFNα^+^ cells are clustered in the HLA-DR intermediate population. Histogram representing 9 animals (**Top**) and dot plot for one representative animal (**Bottom**). (**H**) IFNα and HLA-DR expression on pDC in several compartments on day 10 post-infection showing IFNα^+^ pDC always show HLA-DR intermediate expression level. Dotplots for the two monkeys sacrificed on day 10p.i. are shown (**I**) IFNα production per pDC upon SIV stimulation in 5 infected macaques and 5 uninfected macaques. Paired data sets were compared by Wilcoxon's rank sum test and unpaired data sets by the Mann-Whitney test.

In RhM, the recruitment of pDC to LN during SIV infection is associated with increased pDC death and renewal [27], and with inflammation [9]. In our study, the increased proportion of Ki67^+^pDC in the blood was positively correlated with vRNA loads in both LN (R = 0.72, p = 0.0082) and rectum (R = 0.63, p = 0.026) (Figure 7A); this suggests that tissue vRNA drives the egress of precursors from the BM to the blood to reconstitute the pDC pool in tissues. Statistically significant increases of Ki67^+^ pDC were already observed in LN on day 9 p.i. (Figure 7B), and in other tissues in the two sacrificed animals, on day 10 p.i. (data not shown). We also observed a significant reduction of CD123 expression on pDC in LN on day 9 p.i. (Figure 7C): this may reflect either pDC activation, as we observed decreased CD123 expression after TLR-7/8 stimulation *in vitro* (data not shown), or increased proportions of late-stage pDC precursors [35] that have lost Ki67 expression before homing to LN. To better study the complexity of pDC dynamics in LN, we performed a principal component analysis (APS: Automated Population Separator, using Infinicyt software) of merged files from baseline and day 9 p.i. APS allowed a 2D-plot of pDC events based on the expression levels (MFI) of three markers: CCR7, CD123 and HLA-DR. Three distinct sub-populations in LN were individualized, and named pDC-a, pDC-b and pDC-c (Figure 7D). These populations had distinct phenotypes (Figure 7E): pDC-a displayed a steady-state immature pDC phenotype at baseline (CCR7^low^HLA-DR^int^CD123^high^), pDC-b was CCR7^low^HLA-DR^low^CD123^low^ resembling BM pDC, and pDC-c was CCR7^high^HLA-DR^high^CD123^low/high^, and was most likely activated/matured pDC. The frequency of PDC-a/immature pDC significantly decreased after infection as the pDC-b/HLA-DR^low^ late precursors and pDC–c/activated-matured populations increased (Figure 7F). Although the percentage of pDC-a within total CD45+ LN cells increased significantly (Figure S4), remarkably, the expression of CD123 was significantly decreased on pDC-a at day 9 compared to baseline (p = 0.031). This change in pDCa phenotype indicates that their increase is mainly due to influx of pDC precursors.

IFNα-producing pDC were consistently HLA-DR^int/high^ (Figure 7G and 7H). Very few HLA-DR^low^ pDC, corresponding to BM-pDC precursors (pDC-b), were IFNα^+^, consistent with their pDC precursor profile. Similar patterns were observed in all tissues in which IFNα^+^ pDC were detected (Figure 7H) in samples from the two animals sacrificed on day 10. Biopsies from these macaques at baseline contained sufficient cells for longitudinal monitoring of IFNα production by pDC after SIV stimulation. This production capacity was lower than baseline on day 9 (Figure 7I). These data were then confirmed by a cross-sectional study comparing biopsies from five uninfected macaques to those from five SIV-infected macaques on day 9 (Figure 7I).

These data show that pDC in PLN also loose responsiveness during acute infection concomitant with the increase of pDC sub-populations with no IFNα production ability i.e. pDC precursors and mature pDC. This dynamics likely contributes to the observed rapid decrease of IFNα production in tissues after acute phase, and the rapid disappearance of IFNα from plasma.

## Discussion

This study in CyM revealed several new features of the dynamics of pDC and IFNα production during primary and early chronic infection with pathogenic SIVmac. Our analysis of pDC dynamics confirms that pDC are major contributors to the acute IFNα surge. IFNα production by pDC appeared transient during acute infection and correlates with viral loads in LN and in a wide array of other lymphoid and colorectal tissues. Following a peak of production of IFNα by pDC in tissues and IFNα concentration in the plasma, IFNα production rapidly decreases towards undetectable levels, coinciding with decreased pDC responsiveness *ex vivo*. We show for the first time that the transient impairment of IFNα production by pDC in response to SIV stimulation *ex vivo* is temporally associated and inversely correlated with an increase in the counts of non-functional Ki67^+^ BM-derived pDC in the blood. PDC responsiveness is also impaired in lymph nodes and this is associated with more complex dynamics including increased numbers of both pDC precursors expressing Ki67^+^ and/or low CD123/low HLA-DR, and activated/mature pDC. In addition we show that pDC are strongly activated during the acute infection and this activation persists during chronic infection, although to a lower level than during acute infection.

The major finding of this study is the contribution of the dynamics of pDC sub-populations to the regulation of IFNα production during acute infection. We show that transient impairment of pDC function during acute infection is a consequence of the increase of pDC precursors as a proportion of the circulating pDC pool. Previous studies showed that engagement of TLR-9 [37] or TLR-7 *in vitro* [32] drives pDC to a refractory stage resistant to *de novo* stimulation, and proposed that this could be responsible for pDC unresponsiveness during chronic viral infections [32]. In our model, the ability of circulating pDC to produce IFNα upon *de novo* SIV or HSV stimulation fell sharply (this study and [5]) but this phenomenon was not associated with any sign of pDC activation in this compartment *in vivo*. We showed that the relative increase in the numbers of Ki67^+^ pDC, probably recently egressed from the BM, is most likely to be responsible for this decreased responsiveness. Although BM is also a site of viral replication during SIV infection [38], we did not find any IFNα^+^ pDC in BM. This is consistent with the fact that the pDC pool in both human and RhM BM is mostly constituted of precursor cells which are not yet functional and have only a limited ability to secrete IFNα [35], [36]; here, we confirm that the pDC pool in CyM BM is similar. The peripheral pDC pool becomes exhausted through activation and apoptosis; pDC renewal replenishes this pool, but also regulates the numbers of functional cells, contributing to blunt acute IFNα production rapidly. Although we found a limited increase of early Ki67^+^ pDC precursors in tissues, the numbers of CD123^low^ pDC also increased suggesting recruitment of BM-derived late precursor pDC, which also have limited IFNα production capacities [35]. These precursors were indeed not IFNα^+^ *in vivo*. In parallel, pDC in LN were activated (CCR7^high^ and CD86^+^CD40^+^), but matured cells remained consistently IFNα^−^. The simultaneous increase of these sub-populations in lymphoid tissues coincided with *a decrease* of IFNα^+^ pDC in LN, which most likely contributes to the observed rapid decline of the IFNα concentration in plasma, at a time when virus replication is still high. The rapid blunting of acute IFNα production at around the time of peak viral load may reduce pDC antiviral efficacy, favor the spread of the virus and facilitate the formation of viral reservoirs. Increased pDC renewal during acute infection in our model is associated with massive CD95/Fas death receptor up-regulation on pDC and increased pDC death in all tissues studied, mostly observed during acute infection. Increased apoptosis of pDC has also been reported in HIV-1 infection [21] and is likely a driving force of pDC turnover. In mice, IFNα directly regulates pDC counts *in vivo* by inducing apoptosis [39], and may also regulate conventional DC turnover *in vivo* [40]. Therefore, IFNα may directly regulate both pDC count and pDC function (IFNα production) in our model. However, further investigations are required to determine the details of the mechanisms leading to pDC death *in vivo* in light of a recent report that mDCs are more prone to undergo apoptosis in response to death ligands during acute SIV infection [41].

Another finding in our study is that pDC are strongly activated in PLN during acute infection, displaying CD86 and CD40 co-expression as well as HLA-DR and CCR7 up-regulation. This further implicates pDC in the immune response to HIV/SIV infection. Only partial pDC activation during the chronic stage of HIV-1 infection has been described [21], [42]. The temporal association of this activation pattern with the detection of IFNα-producing pDC suggests that, during acute infection, pDC transiently produce IFNα upon sensing SIV or SIV infected cells, and then acquire other markers of activation and maturation. This complete activation may allow pDC to play a role in priming T-cell responses [43], [44] and contrasts with their partial activation reported after stimulation with inactivated HIV-1 particles *in vitro* [33]. Indeed, another report suggests that stimulation with HIV-infected activated T cells induces a more complete activation phenotype than stimulation with HIV particles *in vitro* [45]. The extent of pDC activation during primary infection suggests that these cells may display other immune functions and orchestrate immune responses in HIV/SIV primary infections, contributing to the cytokine/inflammatory environment, T-cell recruitment and activation in lymph nodes as previously shown in vaginal mucosa [18]. Further work is needed to unravel the complexity of pDC functions during acute and chronic infection.

To conclude, we show for the first time that the dynamics of pDC renewal, which is associated with recruitment, activation and death in lymphoid tissues, reflects the transient decrease of their capacity to produce IFNα in response to SIV during acute infection. Our results shows that this mechanism contributes to pDC unresponsiveness and likely blunt acute IFNα production during primary SIV infection, a mechanism probably transposable to human HIV-1 primary infection in which similar transient pDC deficiency was reported [30]. This transient exhaustion of the anti-viral capacity of the peripheral pDC pool may therefore favor the transition from acute to chronic infection. This new mechanism, which involves pDC renewal in the regulation of IFNα production *in vivo*, may also be relevant to other chronic viral infections when strong pDC activation and death occurs. Further exploration of pDC functions are needed to unravel their complex involvement in early anti-viral immunity and understand their contribution to HIV/SIV induced inflammation and pathogenesis.

## Methods

### Ethics statement

Adult CyM (*Macaca fascicularis*) were imported from Mauritius and housed in the facilities of the “Commissariat à l'Energie Atomique et aux Energies Alternatives” (CEA, Fontenay-aux-Roses, France). Non-human primates (NHP, which includes *M. fascicularis*) are used at the CEA in accordance with French national regulations and under the supervision of national veterinary inspectors (CEA Permit Number A 92-032-02). The CEA complies with the Standards for Human Care and Use of Laboratory Animals, of the Office for Laboratory Animal Welfare (OLAW, USA) under OLAW Assurance number #A5826-01. All experimental procedures were conducted according to European guidelines for animal care (European directive 86/609, “Journal Officiel des Communautés Européennes”, L358, December 18, 1986). The use of NHP at the CEA is also in conformity with the recommendations of the newly published European Directive (2010/63, recommendation N°9). The animals were used under the supervision of the veterinarians in charge of the animal facility. This study was scientifically reviewed and granted by the “Agence Nationale de Recherches sur le SIDA et les hépatites virales” and was accredited under statement numbers 12-007, 12-048 and 12–103, by the ethical committee “Comité d'Ethique en Expérimentation Animale du CEA” registered under number 44 by the French Ministry of Research.

### Animals, infection, and sample collection

Male CyM weighing 5 to 10 kg were used. Fourteen animals were intravenously administered 5,000 animal infectious dose 50% (5,000AID_50_) of the isolate SIVmac251. Virus stock was kindly provided by Dr. A.M Aubertin (Université Louis Pasteur, Strasbourg, France). The experimental design and schedule of sampling are described in Table S1 and Figure S1. Nine of the macaques were subjected to longitudinal follow up: blood, LN and rectal samples were collected from six; and only LN samples were collected from three. Five SIV infected macaques were used for cross sectional sampling: two were sacrificed on day 10 post infection to allow analysis of diverse tissues, and three were sampled on day 9 for cross sectional analysis of pDC responsiveness. Twenty-two naive macaques were used as non-infected controls for cross sectional studies. Blood samples, BM aspirates, and LN and rectal biopsies were collected under general anesthesia by intra-muscular injection of 10 mg/kg ketamine (Rhone-Mérieux, Lyon, France). Blood samples were collected into BD Vacutainer Plus Plastic K_3_EDTA tubes (BD Biosciences, Le Pont de Claix, France). BM aspirates were taken at the iliac crest by punction. Tissue samples were collected in PBS containing 10 µg/mL Brefeldin A (Sigma-Aldritch, St-Louis, MO) or snap frozen in liquid nitrogen for storage at −80°C. Two macaques were sacrificed 10 days after infection by intravenous injection of 180 mg/kg sodium pentobarbital (CEVA santé animale S.A., La ballastiere, France) after general anesthesia with ketamine, and samples of spleen, ascending and descending colon, and mesenteric lymph nodes were harvested.

### Cell preparation

Plasma was isolated from EDTA blood samples by centrifugation for 10 min at 950 g, and cryopreserved at −80°C. Experiments were performed on PBLs or suspensions of single cells extracted from tissue. PBLs were isolated from blood samples after red blood cell lysis by hypotonic shock and washing in PBS. Peripheral and mesenteric lymph node cells, and spleen cells, were obtained by mechanical dissociation using GentleMACS dissociator (Miltenyi Biotech, Paris, France). Suspensions were passed through a 70 µm-pore size cell-strainer, washed with PBS and red blood cells were lysed. Additional density gradient isolation was used for spleen cells before red blood cell lysis. Cell counts were determined with a Vi-CELL (Beckman-coulter, Paris, France). Suspensions of colorectal tissue cells were obtained from sacrificed animals by a protocol used for humans [46] adapted in-house to macaques. Briefly, 1 mm^2^ punches of mucosa were obtained from total ileum, and ascending and descending colon. These samples were treated for 45 min with collagenase II (Sigma-Aldricht) and mechanically disrupted with a 30-mL syringe equipped with an 18-gauge blunt-end needle and passage through a 70-µm-pore cell strainer. Cells were then isolated on a 44%/67.5% Percoll gradient [28].

### Phenotypic characterization and intracellular IFNα staining

PBL isolated from 500 µl of blood or 2–4×10^6^ isolated LN cells were stained for dendritic cells. Aliquots of 50 µl of whole blood were stained for lymphocytes. All stainings, other than of whole blood, were performed after saturation of Fc receptors with healthy macaque serum (in-house production) for 20 min at 4°C. To evaluate cell viability and exclude dead cells from analysis, samples were incubated for 15 min with the amine-reactive dye Live/dead Fixable blue using a commercial dead-cell staining kit (Life technologies). Cells were then labeled with monoclonal antibodies (Table S2) for 15 min at room temperature, washed in PBS and fixed in CellFIX (BD biosciences). Plasmacytoid DCs were gated on CD123^+^HLA-DR^+^ in lineage^−^ cells as previously described [47].

For intra-cellular IFNα labeling, cells isolated from 500 µl of blood or 2–4×10^6^ LN cells were incubated at 37°C for 30 min with 10 µg/mL of Brefeldin A (Sigma-Aldrich). Fc receptor saturation and dead cell staining were performed as described above before intracellular labeling of IFNα. All steps were as previously described [47].

A BD LSRII apparatus equipped with four lasers (355, 405, 488 and 633 nm) was used to acquire data, and data was analyzed with FlowJo v7.6 (Tree Star, Ashland, OR) or Infinicyt v1.6 (Cytognos, Salamanca, Spain) software.

For longitudinal follow-up, acquisition was performed after calibrations with fluorochrome-tagged beads (BD cytometer setup and tracking beads) and using automated application settings. Additionally, fluorescence minus one controls were performed at each time point as intra assay control. For some experiments, flow data were formatted with Pestle v1.6.2 software (Mario Roederer, Vaccine Research Center, National Institute of Allergy and Infectious Diseases, National Institutes of Health) to facilitate the use of SPICE v5.2 [48].

### T-cell and pDC quantification

Absolute counts were calculated from lymphocyte counts obtained by automated cell counting (Coulter MDII; Coultronics, Villepinte, France) combined with flow cytometry data: for lymphocyte counts, the CD4 and CD8 cells as a percentage of the CD45^+^CD3^+^ gate was multiplied by the lymphocyte count. Absolute pDC counts were determined as the product of total leukocyte counts and the percentage of pDC in the CD45^+^ gate.

### *In vitro* stimulation

PBL prepared from 300 µl of blood were cultured for 24 h in RPMI-1640 medium with AT-2-inactivated SIVmac239 (equivalent to 560 ng/mL p27) in a final volume of 200 µl. Supernatants were collected and stored at −80°C until use for IFN-I titration. Negative controls included using the same concentration of AT-2-treated SupT1 micro vesicles. AT-2-inactivated SIVmac239 (ARP1018.1) and its negative control (ARP1018.2) were obtained from Dr Jeff Lifson (National Cancer Institute, Frederick, MD), through the EU Program EVA Centralized Facility for AIDS reagents (National Institute for Biological Standards and Control, Potters Bar, United Kingdom).

### RNA extraction and cDNA synthesis

Tissue lysates were obtained by mechanical disruption of tissue samples in RLT buffer (Qiagen, Courtaboeuf, France) with a Precellys system, using 18 CK tubes and ceramic beads (Bertin Technologies, Montigny-le-Bretonneux, France). Tissue lysates were diluted to 30 mg/mL in RLT buffer, aliquoted and stored at −80°C. Tissue lysates were passed through a QiaShredder (Qiagen) for homogenization, and total RNA was extracted using RNeasy MiniKits (Qiagen) according to the manufacturer's recommendations. To avoid genomic DNA contamination, an additional DNAse step was included after the RNAse-free DNAse Set (Qiagen) used according to the kit instructions. The QuantiTect Rev-Transcription kit (Qiagen) was used to produce cDNA.

### Quantification of type I IFN mRNAs

Quantitative RT-PCR was used to assay IFNα. Briefly, the Quantitect Rev-transcriptase Kit (Qiagen) was used to synthesize cDNA, and pre-PCRs were run in triplicate for each sample using FastStart Taq DNA polymerase (Roche Diagnostics, Meylan, France). A preparation of a plasmid containing macaque IFNα or IFNβ gene of known concentration was amplified during pre-PCR runs as a standard for subsequent qPCR amplification. Quantative PCR was performed with each sample on a Lightcycler using SYBRgreen (Roche) and 0.2 µl of FastStart Taq (Roche) in a final volume of 25 µl. Primers (Table S3) consensus primers for all IFNα subtypes (pan-IFNα in Table S3) were used: pre-PCR (OUT primers) (10 min at 95°C and 21 times (30 s,94°; 30 s,60°C; 4 min,72°C) and qPCR (internal primers) and qPCR (IN primers)(5 min at 95°C and 40 times (10 s,95°; 6 s,60°C; 15 min,72°C) followed by a melting curve). Direct qPCR on extracted RNA with no RT step was used as a negative control for all samples to test for and discard any RNA sample containing genomic DNA. IFN-I activity in plasma was determined with a bioassay measuring the reduction of the cytopathic effect of vesicular stomatitis virus in Madin-Darby bovine kidney cells [49]. The number of surviving cells was determined by MTT dye assay. Antiviral activity is expressed as IC_50_. The IC_50_ was defined as the concentration that was required for 50% protection against VSV-induced cytopathic effects.

### Viral RNA quantification in tissues and plasma

Plasma and tissue vRNA was assayed as previously described [50] and [51] using the primers and probes listed in Tables S3 and S4.

### Data visualization and statistical analysis

Some sets of data were visualized with Tableau Desktop 7.0 software (Tableau Software, Seattle, WA) before statistical analysis. The nonparametric Spearman rank correlation test was used to investigate the relationship between variables. The nonparametric Mann-Whitney test was used to compare groups of macaques after validation with the Kruskall-Wallis test, and the nonparametric Wilcoxon rank sum test was used to compare dependent data (same macaques at different time points) before and after SIV infection. GraphPad Prism 5.03 software (GraphPad software, La Jolla, USA) was used for all statistical analyses. In 2-tailed tests, p values of 0.05 or lower were considered significant.

## Supporting Information

Figure S1 **Schematic diagram of the experimental design.**
(TIF)

Figure S2 **Gating strategy for counting pDC and identification of IFNα^+^ cells.** (**A**) Gating strategy used for counting pDC in PBL. pDC were identified as CD45^+^ HLA-DR^+^ Lineage negative CD123^+^ cells within a morphological SSC^dim/high^ gate after exclusion of doublets and exclusion of dead cells. Backgated pDC are shown in blue. (**B**) Gating strategy used to follow IFNα expression in different cell lineages in PBL. Gating strategy for DC and monocytes: SSC^dim/high^ population, exclusion of CD3^+^ T cells (upper panel), gating on HLA-DR^+^ cells and exclusion of CD20^+^ cells (middle), pDC were identified as CD123^+^, mDCs as CD11c^+^ and monocytes as CD14^+^. Gating strategy for B cells, T cells and NK cells: SSC^low^ population, NK cells were gated as CD3^−^CD8^+^, CD8^+^ T cells as CD3^+^CD8^+^, CD4^+^ T cells as CD3^+^CD8^−^, B cells as CD3^−^CD20^+^HLA-DR^+^. (**C**) Gating strategy used to define DC and macrophage cell populations in lymph nodes. Intracellular staining for IFNα was performed *ex vivo* after 30 min of incubation in 10 µg/ml Brefeldin A with no stimulation.
(TIF)

Figure S3 **Local viral load drives IFNα production by pDC in peripheral lymph nodes.** (**A**) Relative SIVgag mRNA abundance in peripheral lymph nodes at various times after infection. The fast progressor macaque is shown in red and slow progressor in green. ULD = Under the limit of detection. (**B**) Relative IFNα mRNA abundance correlates with relative SIVgag mRNA abundance in peripheral lymph nodes (day 9 p.i., n = 9). Spearman correlation.
(TIF)

Figure S4 **(A) Evolution of the percentage of each pDC subpopulations identified by PCA following SIV infection (from BL to day 9 p.i., n = 9).** (**B**) Changes in CD123 expression levels on pDC-a between baseline and day 9 post-infection.
(TIF)

Table S1 **Timeline of blood sampling and tissue biopsies, in macaques that were infected by SIV.** All sampling time-points are expressed in days except M3 (month 3). n.d: not done. (§) indicates macaques that were sacrificed at 10 days post infection for additional tissue collection including bone marrow, spleen, peripheral lymph nodes, mesenteric lymph nodes, colon, ileum. Lymph nodes from macaques 30602, 30690 and 30044 were used for assaying pDC function only.
(DOCX)

Table S2 **Monoclonal antibodies used for immunophenotyping.** Targeted clusters of differentiation, clones, fluorochromes and commercial origin of antibodies are indicated.
(DOCX)

Table S3 **Sequences of primers used for qPCR.** Sequences of forward and reverse primers used for qPCR are indicated. Primers used for Pre-PCR (OUT) and qPCR (IN) are specified.
(DOCX)

Table S4 **Probes used to quantify SIVgag and GAPDH mRNA expression.** Sequence is given for each probe used for quantification.
(DOCX)
