# Supplementary figures and images for: Plasmacytoid Dendritic Cell Dynamics Tune Interferon-Alfa Production in SIV-Infected Cynomolgus Macaques
Source: PLoS Pathog. 2014 Jan 30;10(1):e1003915. doi: 10.1371/journal.ppat.1003915 (PMC3907389; doi:10.1371/journal.ppat.1003915)

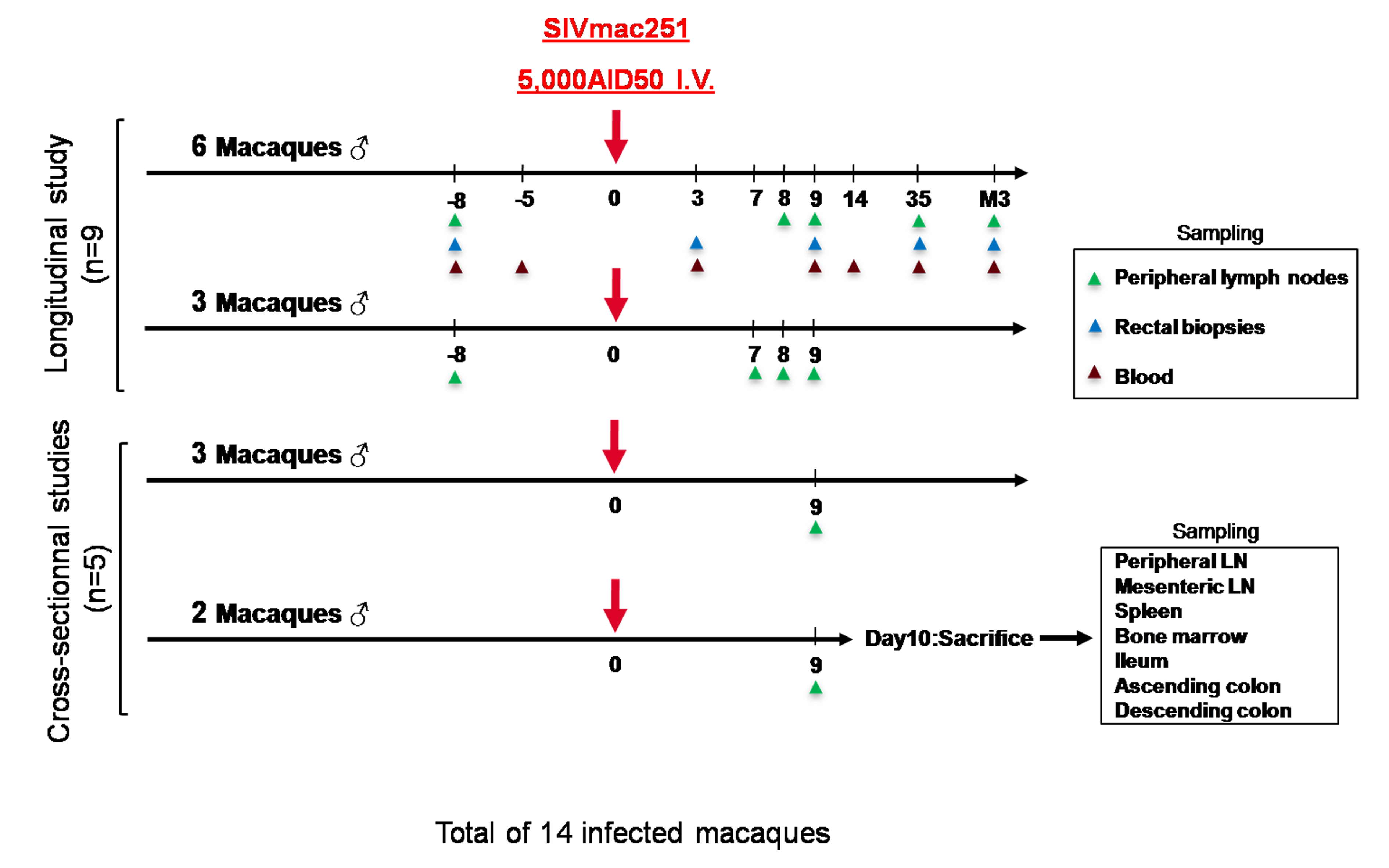

Supplement: Figure S1 — Schematic diagram of the experimental design. (TIF) [file ppat.1003915.s001.tif]

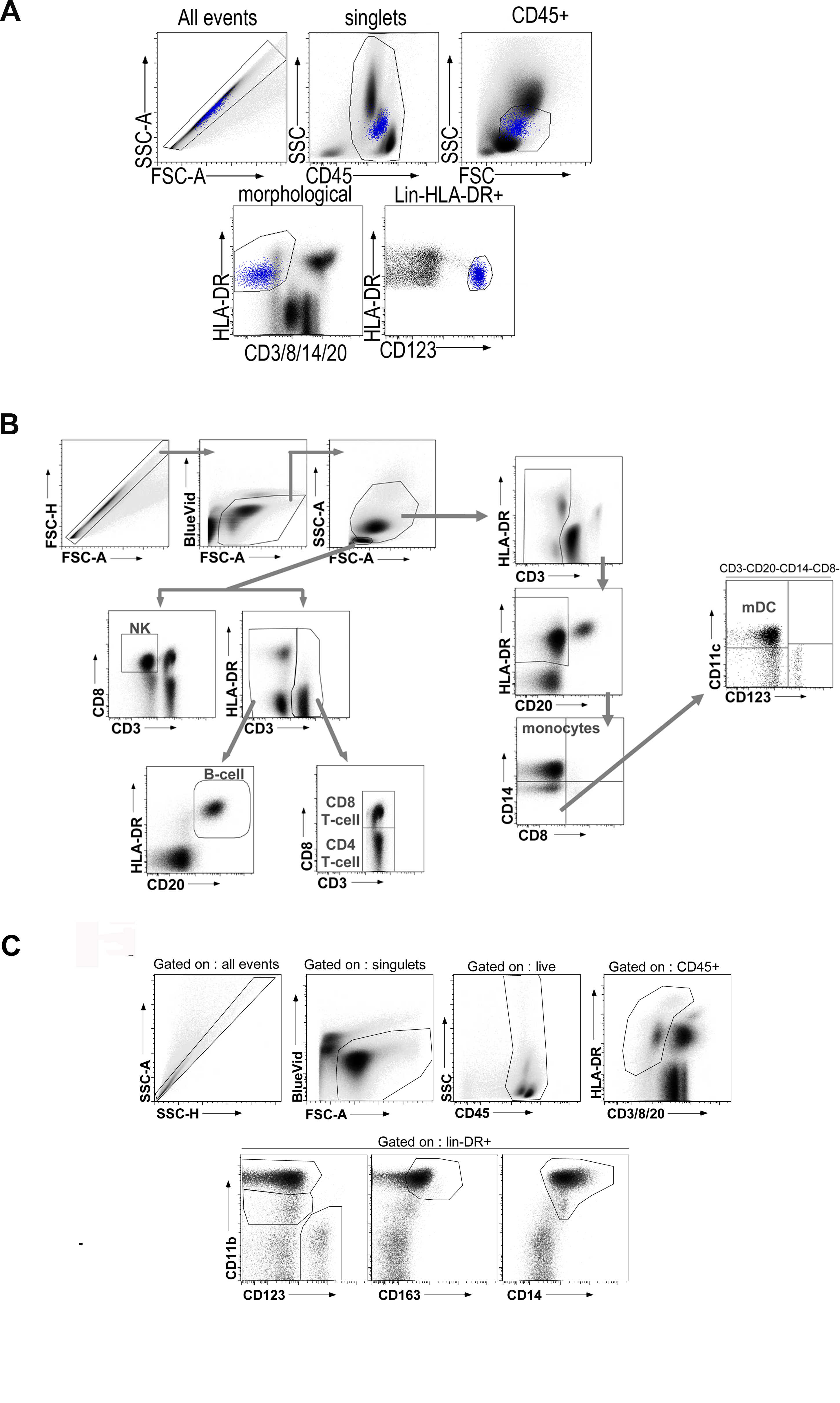

Supplement: Figure S2 — Gating strategy for counting pDC and identification of IFNα+ cells. (A) Gating strategy used for counting pDC in PBL. pDC were identified as CD45+ HLA-DR+ Lineage negative CD123+ cells within a morphological SSCdim/high gate after exclusion of doublets and exclusion of dead cells. Backgated pDC are shown in blue. (B) Gating strategy used to follow IFNα expression in different cell lineages in PBL. Gating strategy for DC and monocytes: SSCdim/high population, exclusion of CD3+ T cells (upper panel), gating on HLA-DR+ cells and exclusion of CD20+ cells (middle), pDC were identified as CD123+, mDCs as CD11c+ and monocytes as CD14+. Gating strategy for B cells, T cells and NK cells: SSClow population, NK cells were gated as CD3−CD8+, CD8+ T cells as CD3+CD8+, CD4+ T cells as CD3+CD8−, B cells as CD3−CD20+HLA-DR+. (C) Gating strategy used to define DC and macrophage cell populations in lymph nodes. Intracellular staining for IFNα was performed ex vivo after 30 min of incubation in 10 µg/ml Brefeldin A with no stimulation. (TIF) [file ppat.1003915.s002.tif]

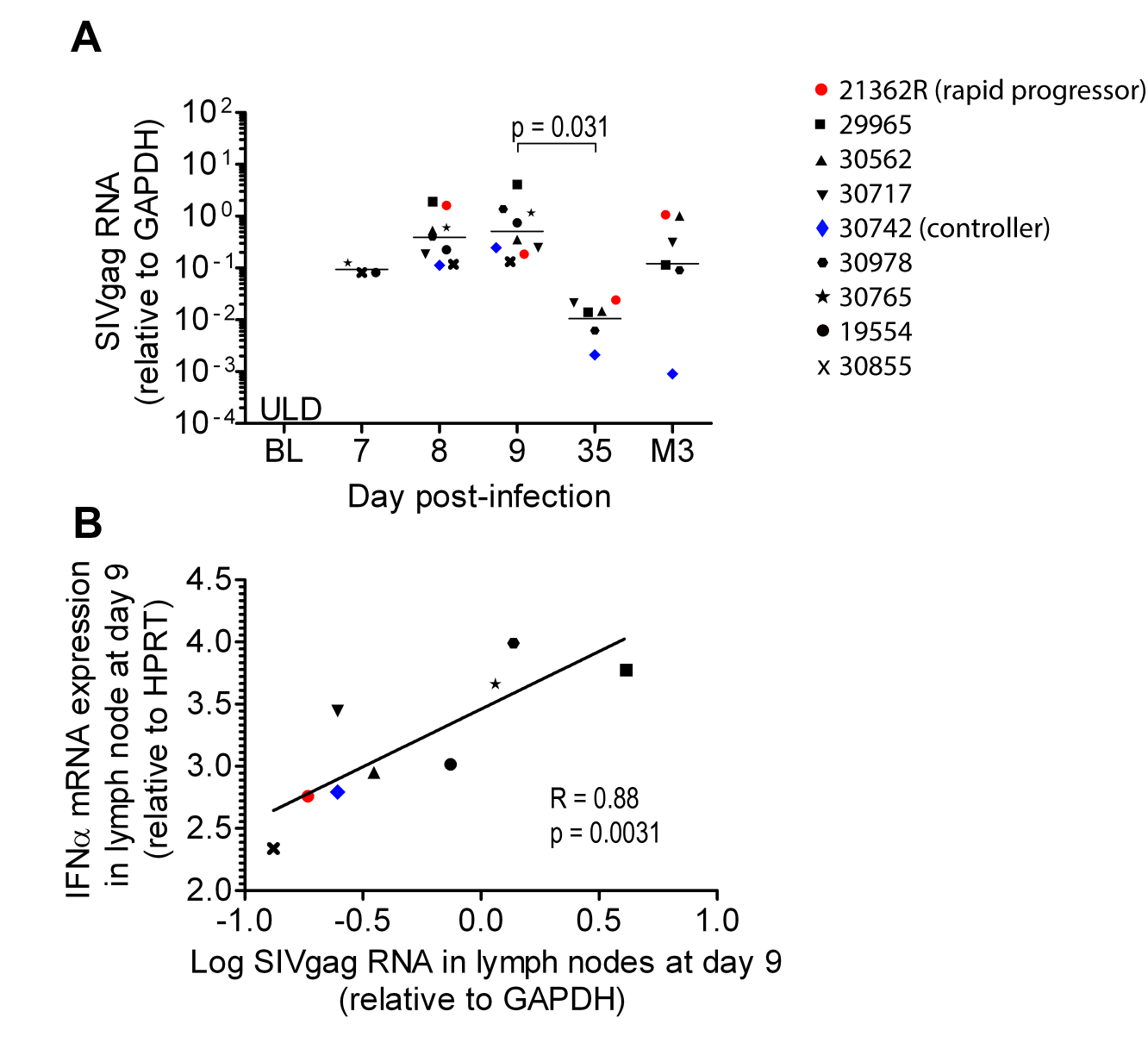

Supplement: Figure S3 — Local viral load drives IFNα production by pDC in peripheral lymph nodes. (A) Relative SIVgag mRNA abundance in peripheral lymph nodes at various times after infection. The fast progressor macaque is shown in red and slow progressor in green. ULD = Under the limit of detection. (B) Relative IFNα mRNA abundance correlates with relative SIVgag mRNA abundance in peripheral lymph nodes (day 9 p.i., n = 9). Spearman correlation. (TIF) [file ppat.1003915.s003.tif]

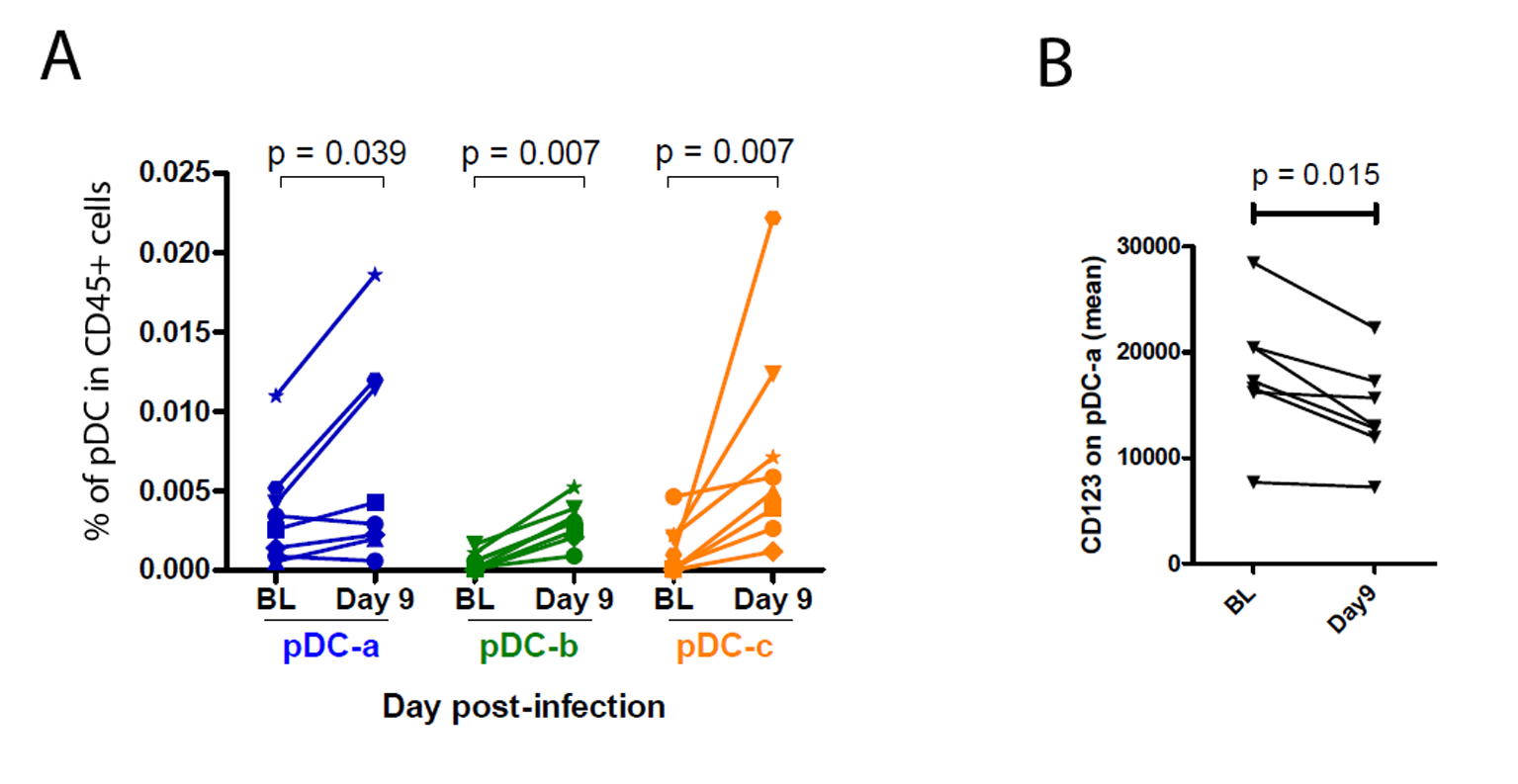

Supplement: Figure S4 — (A) Evolution of the percentage of each pDC subpopulations identified by PCA following SIV infection (from BL to day 9 p.i., n = 9). (B) Changes in CD123 expression levels on pDC-a between baseline and day 9 post-infection. (TIF) [file ppat.1003915.s004.tif]
