# Supplementary material for: Plasmacytoid Dendritic Cell Dynamics Tune Interferon-Alfa Production in SIV-Infected Cynomolgus Macaques
Source: PLoS Pathog. 2014 Jan 30;10(1):e1003915. doi: 10.1371/journal.ppat.1003915 (PMC3907389; doi:10.1371/journal.ppat.1003915)
Supplement: Table S1 — Timeline of blood sampling and tissue biopsies, in macaques that were infected by SIV. All sampling time-points are expressed in days except M3 (month 3). n.d: not done. (§) indicates macaques that were sacrificed at 10 days post infection for additional tissue collection including bone marrow, spleen, peripheral lymph nodes, mesenteric lymph nodes, colon, ileum. Lymph nodes from macaques 30602, 30690 and 30044 were used for assaying pDC function only. (DOCX) [file ppat.1003915.s005.docx]

**Table S1:**

| **Macaque** | **Blood** | **Lymph nodes** | **Rectal Biopsies** |
| --- | --- | --- | --- |
| 21362R | -8,-5, 3, 9, 14, 35, M3 | -8, 8, 9, 35, M3 | -8, 3, 9, 35, M3 |
| 29965 | -8,-5, 3, 9, 14, 35, M3 | -8, 8, 9, 35, M3 | -8, 3, 9, 35, M3 |
| 30562 | -8,-5, 3, 9, 14, 35, M3 | -8, 8, 9, 35, M3 | -8, 3, 9, 35, M3 |
| 30717 | -8,-5, 3, 9, 14, 35, M3 | -8, 8, 9, 35, M3 | -8, 3, 9, 35, M3 |
| 30742 | -8,-5, 3, 9, 14, 35, M3 | -8, 8, 9, 35, M3 | -8, 3, 9, 35, M3 |
| 30978 | -8,-5, 3, 9, 14, 35, M3 | -8, 8, 9, 35, M3 | -8, 3, 9, 35, M3 |
| 30765 | n.d | -8, 7, 8, 9 | n.d |
| 19554 | n.d | -8, 7, 8, 9 | n.d |
| 30855 | n.d | -8, 7, 8, 9 | n.d |
| 21175R(§) | n.d | 9 | n.d |
| 31047(§) | n.d | 9 | n.d |
| 30602 | n.d | 9 | n.d |
| 30690 | n.d | 9 | n.d |
| 31044 | n.d | 9 | n.d |
