# Supplementary material for: Plasmacytoid Dendritic Cell Dynamics Tune Interferon-Alfa Production in SIV-Infected Cynomolgus Macaques
Source: PLoS Pathog. 2014 Jan 30;10(1):e1003915. doi: 10.1371/journal.ppat.1003915 (PMC3907389; doi:10.1371/journal.ppat.1003915)
Supplement: Table S2 — Monoclonal antibodies used for immunophenotyping. Targeted clusters of differentiation, clones, fluorochromes and commercial origin of antibodies are indicated. (DOCX) [file ppat.1003915.s006.docx]

**Table S2 :**

| **Cluster** | **Clone** | **Fluorochrome** | **Origin** |
| --- | --- | --- | --- |
| CD3 | SP34-2 | AF700 | BD Biosciences |
| CD3 | FN18 | FITC | Invitrogen |
| CD3 | SP34-2 | v450 | BD Biosciences |
| CD4 | L200 | PE | BD Biosciences |
| CD8 | RPA-T8 | PE-Cy7 | BD Biosciences |
| CD8 | DK-25 | FITC | Dako |
| CD8 | RPA-T8 | Pacific Blue | BD Biosciences |
| CD8 | RPA-T8 | v450 | BD Biosciences |
| CD11b | Bear-1 | PE-Cy7 | Beckman Coulter |
| CD11c | S-HCL-3 | APC | BD Biosciences |
| CD14 | M5E2 | FITC | BD Biosciences |
| CD14 | M5E2 | v450 | BD Biosciences |
| CD20 | B9E9 | PE-Cy5 | Beckman Coulter |
| CD20 | B9E9 | FITC | Beckman Coulter |
| CD20 | L27 | v450 | BD Biosciences |
| CD28 | CD28.2 | ECD | Beckman Coulter |
| CD34 | 563 | PE | BD Biosciences |
| CD38 | AT-1 | FITC | StemCell |
| CD40 | 5C3 | PE-Cy7 | BD Biosciences |
| CD45 | D058-1283 | PerCP | BD Biosciences |
| CD45 | D058-1283 | V450 | BD Biosciences |
| CD86 | 2331(FUN-1) | PE | BD Biosciences |
| CD95 | DX2 | APC | BD Biosciences |
| CD123 | 7G3 | PercP-Cy5.5 | BD Biosciences |
| CD163 | GHI/61 | PE | Ozyme |
| CD197 (CCR7) | 150503 | PE | R&D system |
| HLA-DR | L243 | APC-H7 | BD Biosciences |
| IFNα | LT27 :295 | PE | Miltenyi Biotech |
| Ki67 | MIB-1 | FITC | Dako |
| Ki67 | B56 | AF700 | BD Biosciences |
