# Supplementary material for: Plasmacytoid Dendritic Cell Dynamics Tune Interferon-Alfa Production in SIV-Infected Cynomolgus Macaques
Source: PLoS Pathog. 2014 Jan 30;10(1):e1003915. doi: 10.1371/journal.ppat.1003915 (PMC3907389; doi:10.1371/journal.ppat.1003915)
Supplement: Table S3 — Sequences of primers used for qPCR. Sequences of forward and reverse primers used for qPCR are indicated. Primers used for Pre-PCR (OUT) and qPCR (IN) are specified. (DOCX) [file ppat.1003915.s007.docx]

**Table S3 :**

| **Target** | | **Forward** | | **Reverse** |
| --- | --- | --- | --- | --- |
| SIV-gag | | GCAGAGGAGGAAATTACCCAGTAC | CAATTTTACCCAGGCATTTAATGTT |  |
| GAPDH | | GAAGGTGAAGGTCGGAGTC | GAAGATGGTGATGGGATTTC |  |
| Pan-IFNα OUT | | TCTCCTGCCTGAAGGACAGA | TCTCATGATTTCTGCTCTGACA |  |
| Pan-IFNα IN | | AAGCCATCTCTGTCCTCCAT | ATGATTTCTGCTCTGACAACCT |  |
| HPRT OUT | | CTGAACGTCTTGCTCGAGAT | CGACCTTGACCATCTTTGGA |  |
| HPRT IN | | CACATTGTAGCCCTCTGTGT | CTGACCAAGGAAAGCAAAGT |  |
