# Supplementary material for: Plasmacytoid Dendritic Cell Dynamics Tune Interferon-Alfa Production in SIV-Infected Cynomolgus Macaques
Source: PLoS Pathog. 2014 Jan 30;10(1):e1003915. doi: 10.1371/journal.ppat.1003915 (PMC3907389; doi:10.1371/journal.ppat.1003915)
Supplement: Table S4 — Probes used to quantify SIVgag and GAPDH mRNA expression. Sequence is given for each probe used for quantification. (DOCX) [file ppat.1003915.s008.docx]

**Table S4 :**

| **Target** | **Sequence** |
| --- | --- |
| SIVgag | TGTCCACCTGCCATTAAGCCCGA |
| GAPDH | CAAGCTTCCCGTTCTCAGCC |
